# Functionalized Carbon Nanotube and MnO_2_ Nanoflower Hybrid as an Electrode Material for Supercapacitor Application

**DOI:** 10.3390/mi12020213

**Published:** 2021-02-20

**Authors:** Sagar Mothkuri, Honey Gupta, Pawan K. Jain, Tata Narsinga Rao, Gade Padmanabham, Supriya Chakrabarti

**Affiliations:** 1 Centre for Carbon Materials, International Advanced Research Centre for Powder Metallurgy and New Materials, Hyderabad, P.O. Balapur, Telangana 500005, India; sagarmothkuri8@gmail.com (S.M.); egineer.honey.gupta@gmail.com (H.G.); pkjain@arci.res.in (P.K.J.); tata@arci.res.in (T.N.R.); gp@arci.res.in (G.P.); 2 Nanotechnology and Integrated Bio-Engineering Centre (NIBEC), School of Engineering, Ulster University, Newtownabbey BT37 0QB, UK

**Keywords:** FCNT-MnO_2_, hydrothermal, supercapacitor, specific energy, specific power

## Abstract

Functionalized carbon nanotube (FCNT) and Manganese Oxide (MnO_2_) nanoflower hybrid material was synthesized using hydrothermal technique as a promising electrode material for supercapacitor applications. The morphological investigation revealed the formation of ‘nanoflower’ like structure of MnO_2_ connected with FCNT, thus paving an easy path for the conduction of electrons during the electrochemical mechanism. A significant improvement in capacitance properties was observed in the hybrid material, in which carbon nanotube acts as a conducting cylindrical path, while the major role of MnO_2_ was to store the charge, acting as an electrolyte reservoir leading to an overall improved electrochemical performance. The full cell electrochemical analysis of FCNT-MnO_2_ hybrid using 3 M potassium hydroxide (KOH) electrolyte indicated a specific capacitance of 359.53 F g^−1^, specific energy of 49.93 Wh kg^−1^ and maximum specific power of 898.84 W kg^−1^ at 5 mV s^−1^. The results show promise for the future of supercapacitor development based on hybrid electrode materials, where high specific energy can be achieved along with high specific power and long cycle life.

## 1. Introduction

The depleting climate conditions and near extinction of fossil fuels have significantly demanded a shift from the non-renewable energy resources to renewable energy sources. The various renewable energy sources such as hydel, solar, wind, biomass, geothermal, and waves-and-tides are geographically and seasonally limited and are not the continuous sources of power generation [1]. Hence, generating and storing renewable energy is the motto of the future research [2]. The most commonly available energy storage devices are capacitors, batteries, and supercapacitors. Of these, the supercapacitor is the energy storage device characterized by being fast charging-discharging, and with long cycle life with considerable energy density as that of batteries [3]. Based on their mechanism, supercapacitors are classified into two foremost types, electric double layer capacitor (EDLC) and pseudo-capacitor [4,5,6]. While, a third classification includes the combination of both EDLC and pseudo materials and is termed ‘hybrid’ supercapacitor [7,8,9,10]. EDLC involves carbon-based materials [11,12] such as activated carbon [13], carbon nanotubes [14], and reduced graphene oxide/graphene [15,16,17] as active electrode materials, while the pseudo-capacitor is based on metal-oxides [18], -hydroxides [19], -sulfides [20], -nitrides [21], and conducting polymers [22,23]. Different morphologies of MnO_2_ such as nanorods, flakes, and nanoflowers were grown on the surface of CNT in previous studies [24]. But the morphological observation shows that the nanoflowers (with flakes) grown on the surface of CNT along its length is convincing to contribute to higher surface area, and in turn to high specific energy and CNT as a fast-conducting path, leading to high specific power. Hence, the current work is directed towards growing MnO_2_ nanoflower morphology on the surface of functionalized carbon nanotubes (FCNT) to obtain high energy density electrode.

Achieving higher specific energy in supercapacitor is a challenge. The incorporation of pseudo materials such as metal oxides can be a good option to achieve high specific energy as well as high specific power, since they have a high theoretical surface area and high charge storage capability [7,8,25]. Among various pseudo materials, MnO_2_ has gained significant importance in recent times due to its existence in various nanoforms with high supercapacitance [26,27,28,29]. It has a high theoretical specific capacitance [30]. It has shorter diffusion ion lengths than its bulk counterpart [31]. Its tunnel like crystal structure is unique in terms of its application as a high charge storage material. These tunnels result in the low density of the material, high permeation of the cations during electrochemical mechanism, and storage of cations of the electrolyte e.g., K^+^, Li^+^, Na^+^ etc. [32]. Besides these exquisite features, its low cost, abundance, excellent redox mechanism, high energy density, and eco-friendliness are other notable features [33,34,35]. However, its low ion diffusion constant and low electronic conductivity are the primary limitations from being a good electrode material by itself for supercapacitor. 

In the present work, we have successfully synthesized MnO_2_ nanoflowers interconnected by FCNT, to cater the enhanced surface area of this particular flower like morphology of MnO_2_ and high electrical conductivity of carbon nanotubes for achieving high energy density supercapacitor. Both MnO_2_ and FCNT-MnO_2_ hybrid have been synthesized using hydrothermal technique. This hydrothermal synthesis method has been adopted because of its simplicity and easy scalability [24]. A detail analysis on the development of morphology and its effect on the enhancement of electrochemical performance are discussed. Comparative electrochemical analysis is done for FCNT, MnO_2_, and FCNT-MnO_2_ hybrid. Supercapacitor devices have been fabricated using this hybrid as electrode material and found to have superior performances like high specific capacitance, fast charge-discharge, long cycle life, high specific power, and high specific energy comparable to batteries.

## 2. Materials and Methods

All the chemicals used in this work are of analytical grade and used without further purification.

### 2.1. Materials 

The multiwalled carbon nanotubes (CNTs) were grown by catalyst assisted chemical vapor deposition (CVD) technique. Hydrochloric acid (HCl) (35.4%) (SDFCL), Nitric acid (HNO_3_) (69–72%) (SDFCL), Sulfuric acid (H_2_SO_4_) (98%) (SDFCL), and Potassium Permanganate (KMnO_4_) extra-pure (SRL) were used for this work.

### 2.2. Functionalization of CNT 

The as synthesized CVD-CNTs were oxidized at 350 °C for an hour in the presence of air to remove amorphous carbon, followed by the 31 wt% HCl treatment for 18 h. The HCl treatment was done to remove the embedded Fe catalysts from the CNTs [36]. Later the HCl treated CNTs were vacuum filtered and washed several times with deionized water to bring down the pH to 7, after which the resultant product was refluxed at 180 °C for 4 h in the presence of 10 M HNO_3_ in a reflux-condenser system. The HNO_3_ treatment was carried out to generate functional groups like hydroxyl and carboxyl groups on the surface of CNTs [37]. The functionalized carbon nanotubes (FCNT) were vacuum filtered several times with deionized water to bring pH to 7 and dried overnight in a vacuum oven at 90 °C. 

### 2.3. Synthesis of MnO_2_

First, 0.6 g of KMnO_4_ was dissolved in 50 mL deionized water and stirred vigorously until a homogeneous solution was formed. During stirring, 0.5 mL of 98 wt% conc. H_2_SO_4_ was added and continued stirring for another 15 min. Then, this homogeneous solution was then transferred into a stainless-steel Teflon lined autoclave and heat treated at 160 °C for 4 h. Finally, a light brownish color precipitate was collected, washed, filtered and vacuum dried overnight at 90 °C. 

### 2.4. Synthesis of FCNT-MnO_2_ Hybrid 

The FCNT dispersion in the 50 mL aqueous solution was prepared using ultra sonication. Later KMnO_4_ (FCNT: KMnO_4_ = 1:3 weight ratio) was added to the above nanotube suspension and then stirred vigorously using magnetic stirrer for an hour. The above mixture was then transferred into Teflon-lined stainless-steel autoclave. The autoclave was subjected to hydrothermal process at 160 °C for 6 h. The solution obtained after the hydrothermal process was washed multiple times using distilled water. Finally, the hybrid material was collected using vacuum filtration and then dried overnight in the vacuum oven at 90 °C.

### 2.5. Characterization

The following section briefs out the characterization techniques utilized in the current work. 

The functional groups of FCNT were investigated by Fourier-transform infrared spectroscopy (FTIR) BRUKER Alpha II (Billerica, MA, USA). The sample for the analysis was prepared using 1:200 of FCNT: KBr for making a transparent pellet. The crystallographic information of FCNT, MnO_2_, and FCNT-MnO_2_ were investigated using D8 Advance, Bruker, US, Powder X-Ray Diffractometer employing Cu Kα radiation with a wavelength of 1.54056 Å at a scan rate of 1.2° min^−1^ (step size of 0.02 deg s^−1^). The fine powder sample of each material was used for XRD analysis. The morphological analysis of FCNT, MnO_2_, and FCNT-MnO_2_ were done using field emission scanning electron microscopy (Gemini SEM 500, ZEISS, Oberkochen, Germany). The sample for the analysis was in the fine powder form. The energy dispersive x-ray spectroscopy (EDS) of the samples was conducted in addition to the morphological analysis (FESEM). Transmission electron microscopy (TEM) was carried out using a TECNAI G2 (FEI Company, Hillsboro, OR, USA) equipped with a field emission electron gun operated at 200 kV. First the powder sample was dissolved in isopropyl alcohol using ultrasonication to get uniform dispersion of the materials and then was directly dropped onto a holey carbon coated Cu grid of 300 meshes and evaporated overnight to prepare the samples for TEM study. Finally, the electrochemical analysis of the materials was done for the application part as a supercapacitor using BioLogic Science Instruments. The analysis was conducted using three techniques: cyclic voltammetry (CV), galvanostatic charge discharge (GCD), and potentiostatic electrochemical impedance spectroscopy (PEIS or EIS, BioLogic, Seyssinet-Pariset, France). The cell preparation details (Figure S9), analysis input parameters and the analytical formulae used are given in the supplementary document.

## 3. Results and Discussion

In this work the MnO_2_ nanoflowers and FCNT-MnO_2_ hybrid material was successfully synthesized using the facile hydrothermal method. The as-received CNTs were functionalized in order to supplement the CNT surfaces with –OH and –COOH functional groups. As prepared CNTs have limited chemical interactions with other compounds and they tend to agglomerate due to high van der Waals force between the tube surfaces. The functionalization of CNTs can alter the surface energy and attached carboxylic or hydroxyl functional groups can improve their chemical reactivity and compatibility [9,38]. The –OH and –COOH functional groups help in the binding of pseudo material MnO_2_ on to the surface of FCNT. The chemical functionalization methods to modify the carbon nanotube surface chemistry [39] by attaching carboxylic or hydroxyl functional groups was evidenced by the FTIR spectrum (Figure S1). The functionalization helped CNT surfaces to become nucleation sites for MnO_2_ and facilitate easy binding among the CNT and MnO_2_. Without functionalization, the CNT and MnO_2_ might stay separate and the hybrid structure will have the possibility to become a mixture of individual constituents. In this work, we aimed to have a hybrid structure where CNT and MnO_2_ should be interconnected to each other so that we get superior capacitive property. 

The formation mechanism of FCNT-MnO_2_ hybrid was primarily driven by nucleation and the successive guided crystal growth of MnO_2_ along the surface of carbon nanotube. In this process, the surface of FCNT was supposedly acting as the nucleation site leading to the limited aggregation of MnO_2_ nanocrystallites. During the synthesis, as the aggregation proceeds, it leads to the formation of nanorods which coalesce to form nanoflowers [40,41,42]. In this work, conc. H_2_SO_4_ merely serveed the purpose of an oxidizing agent during the synthesis of MnO_2_. Formation of MnOOH was commonly observed during the synthesis of MnO_2_ from KMnO_4_ using hydrothermal method [43,44]. Conc. H_2_SO_4_ helped in preventing the formation of the needle like growth of MnOOH during hydrothermal synthesis of MnO_2_. During the synthesis of FCNT-MnO_2_, conc. H_2_SO_4_ was not used because in this synthesis procedure, the heterogeneous nucleation takes place, and there was no scope for the formation of MnOOH as the growth dynamics of MnO_2_ was restricted to the surface of FCNT [45]. The addition of conc. H_2_SO_4_ to FCNTs will result in the longitudinal cleavage of the multi-walled structure [45,46] during the hydrothermal synthesis, which might further damage the carbon nanotubes structure and reduce the electrical conductivity. As such, conc. H_2_SO_4_ was not used during the synthesis of hybrid FCNT-MnO_2_. The reaction mechanisms [24] for the synthesis of MnO_2_ and FCNT-MnO_2_ are given below in Equations (1) and (2):

For MnO_2_:

(1) $$4{\mathrm{MnO}}_{4}^{-}+\;2H_{2}O\;\to 4{\mathrm{MnO}}_{2}+4{\mathrm{OH}}^{-}+3O_{2}$$

For FCNT-MnO_2_:

(2) $$4{\mathrm{MnO}}_{4}^{-}+3C+H_{2}O\to 4{\mathrm{MnO}}_{2}+{\mathrm{CO}}_{3}^{2-}+2{\mathrm{HCO}}_{3}^{-}$$

The initial stirring of FCNT with KMnO_4_ resulted in the formation of MnO_2_ nano crystallites on the surface of FCNT at room temperature. Later, during the hydrothermal process, the MnO_2_ nano crystallites coalesce together during the nucleation, and then further grow into flakes which appeared as nanoflowers on whole with FCNT acting as interconnectors in the hybrid material formation [24].

**Figure 1:**
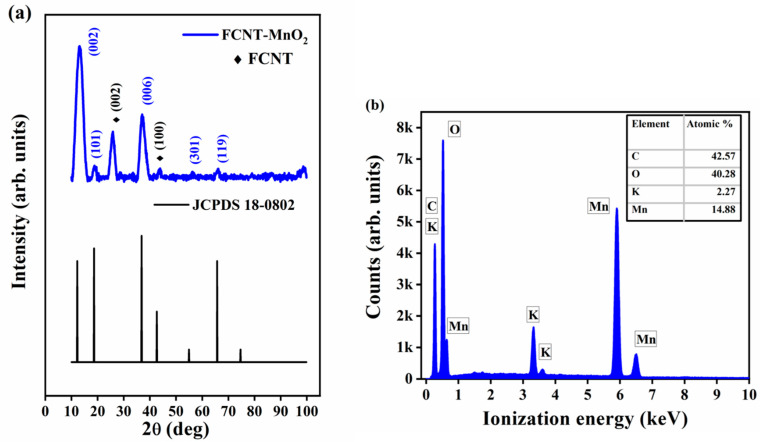
(**a**) XRD pattern of FCNT-MnO_2_ and (**b**) energy dispersive x-ray (EDS) spectrum of FCNT-MnO_2_.

The crystal structural information was investigated by XRD and is shown in the Figure 1a. From the XRD patterns, it is evident that the structure of the functionalized CNT (FCNT) is well preserved even after the functionalization with its two main diffraction peaks at 25.8° (002) and 43.5° (100) belonging to hexagonal crystal system, matching with JCPDS 41-1487 (Figure S2a). The d-spacing was found to be 0.342 nm at (002) plane of FCNT. MnO_2_ has polycrystalline nature with tetragonal crystal system, matching with JCPDS 72-1982 and the d-spacing was found to be 0.673 nm at 12.71° (110) with average crystallite size of 3.17 nm (Figure S2b). The narrow sharp peaks in MnO_2_ indicated high crystallinity. In the hybrid nanostructure of FCNT-MnO_2_, the XRD diffraction pattern resulted from the two diffraction patterns. The peaks (110) at 12.8°, (200) at 18.67°, (121) at 37.3°, (600) at 56.15° and (161) at 66.09° resulted from the pure MnO_2_ birnessite phase, and other two peaks at 25.8° (002) and 43.6° (100) resulted from FCNT. The hybrid showed a tetragonal structure of MnO_2_ which was in good agreement with JCPDS number 72-1982. The small intensity in the peaks related to FCNT indicated that FCNT was partly covered with MnO_2_ and that the majority of the hybrid material was MnO_2_ with fewer FCNTs. The d-spacing resulting from the hybrid material (due to MnO_2_) at 12.8° (110) plane was found to be 0.673 nm. The small peak around 99° (Figure 1a) might have appeared from the aluminum sample holder of XRD (Al metal (400) plane). 

The elemental analysis using EDS of the hybrid, shown in the Figure 1b confirmed the presence of C, O, Mn, and few traces of K. These minute traces of K could be due to the mineral source of manganese salt. We found that there was a non-stoichiometric ratio between Mn and O (1:2.7) which could be attributed to the morphological influence of FCNT during the growth mechanism of the hybrid nanostructure [13]. The elemental analysis of FCNT and MnO_2_ shown in the supporting information (Figure S2c,d respectively) showed that FCNT consisted of carbon and little amount of oxygen occurring due to the presence of functional groups on the surface of the nanotubes, while MnO_2_ had maintained near stoichiometric ratio of 1:1.85 between them with very few traces of K. 

**Figure 2:**
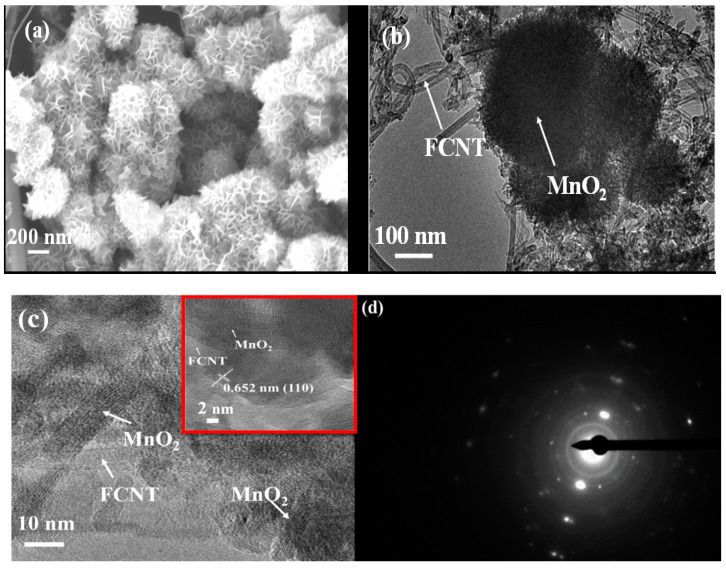
(**a**) FESEM of FCNT-MnO_2_, (**b**) TEM analysis of FCNT-MnO_2_, (**c**) HRTEM of FCNT-MnO_2_ with inset showing the lattice spacing of MnO_2_ in the hybrid FCNT-MnO_2_, and (**d**) SEAD pattern of FCNT-MnO_2_.

The morphology of FCNT-MnO_2_ hybrid was obtained from FESEM analysis. The nanoflower-like morphology was observed for FCNT-MnO_2_ hybrid as shown in the Figure 2a. An average flake void of 1.54 nm between the petals of the nanoflower could be useful as the reservoir for the electrolyte thus enhancing the specific surface area contribution. The morphology of FCNT (entangled) and MnO_2_ (nanoflakes) are given in the supporting information (Figure S3a,b respectively). The change of MnO_2_ nanoflakes to nanoflowers could be due to the influence of FCNT surface on the MnO_2_ growth mechanism [24,47,48]. The average flake size of MnO_2_ was found to be 25–35 nm (Figure S3b, analyzed by ImageJ). The nanoflower-like structure helps in the easy intercalation-deintercalation of the electrolyte ions. This hybrid nanostructure is advantageous for the considerable energy density contributed from MnO_2_ with a decrement in the overall resistance of the hybrid material.

The TEM analysis of FCNT-MnO_2_ is presented in Figure 2b,c. The diameter of the FCNT was calculated using ImageJ analysis of TEM micrographs and was found to be varying between 15 nm and 25 nm with majority of nanotubes having a diameter of 20 nm. It could also be seen from Figure 2b that certain MnO_2_ nanoflowers grew on the surface of FCNT. The MnO_2_ nanoflowers were observed as dark flowers on the surface of entangled carbon nanotubes. The high resolution TEM image is shown in Figure 2c, which indicates that FCNT and MnO_2_ lattice fringes overlapping onto each other. This proves that the MnO_2_ grew on the surface of FCNT. The numbers of walls of FCNT were found to be 12 to 15 with lattice spacing of 0.34 nm. The lattice spacing of MnO_2_ was found to be 0.652 nm for (110) plane as shown in the inset of Figure 2c which is close to the d-spacing calculated from the X-ray diffraction pattern of FCNT-MnO_2_. The SAED pattern shown in the Figure 2d indicates a series of rings with bright spots indicating that the hybrid material consisted of well crystalline MnO_2_ with tetragonal crystal structure. As shown in the SAED pattern, the rings correspond to the (301), (400), and (220) planes of tetragonal MnO_2_ and matching well with that of XRD pattern of MnO_2_ (Figure S2b).

The FESEM and TEM images (Figure 2) of FCNT-MnO_2_ hybrid indicates the growth of MnO_2_ onto FCNT surface. Figure 2a,b clearly show nanoflower like morphology of MnO_2_ on FCNT surface similar like seen in previous published results [24,45]. The evidence of the coupling between CNT and other material in the hybrid can be seen from the results of EDS, FESEM, and TEM in other published literatures like Bi_2_S_3_@CNT [49], Ag/CNT [50], CNT-MoS_2_ [9] and CNT-MnO_2_ [51]. Besides that, Figure 2c reveals the high resolution TEM image of FCNT-MnO_2_, which confirms two different lattice-spacings that belong to FCNT and MnO_2_ within a given area. From the high resolution TEM image (Figure 2c), it can be clearly observed that FCNT and MnO_2_ lattices are interconnected and overlapped to each other. This is a general feature observed in HRTEM images from different portions of the sample, thus it can be claimed that FCNT surface acted as nucleation sites for MnO_2_. Also, the classical nucleation theory says that heterogeneous nucleation is much more common in hybrid nanostructure and the nucleation happens at the interfaces and surface imperfections [49,50,51,52]. The Raman spectrum (Supporting information Figure S4) of FCNT-MnO_2_ indicates a D-Band or structural defect dominated structure of FCNT, and these defect sites of FCNT work as nucleation sites for MnO_2_ as per heterogeneous nucleation mechanism [53]. The delayered graphene sheets from multi-walled structure of FCNT may also act as nucleation sites during the hydrothermal synthesis of FCNT-MnO_2_ hybrid [54]. Additionally, the functionalized CNT surface with attached COOH and OH functional groups are favorable of chemical reactivity and provide suitable binding ligands for MnO_2_ during the growth. Another factor is activation energy, which plays a key role in the growth of hybrid nanostructures [54,55]. The high surface area of FCNT decreases the activation energy required for the nucleation of the MnO_2_, it subsides the complete growth of MnO_2_ nanoflowers. Henceforth, the size of the MnO_2_ nanoflower is significantly reduced after composite which is clearly evidenced from the comparison of Figure 2a and Figure S3b. These results stand as an evidence for the growth of MnO_2_ onto FCNT surface.

**Figure 3:**
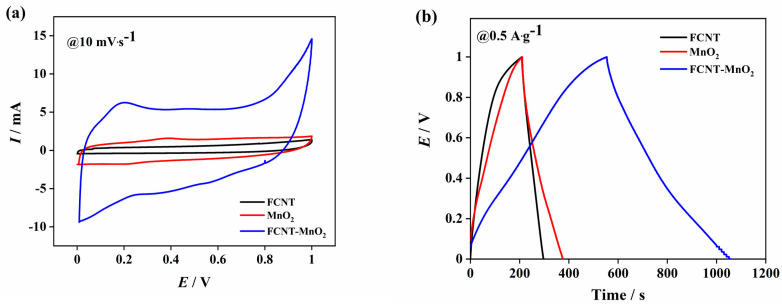

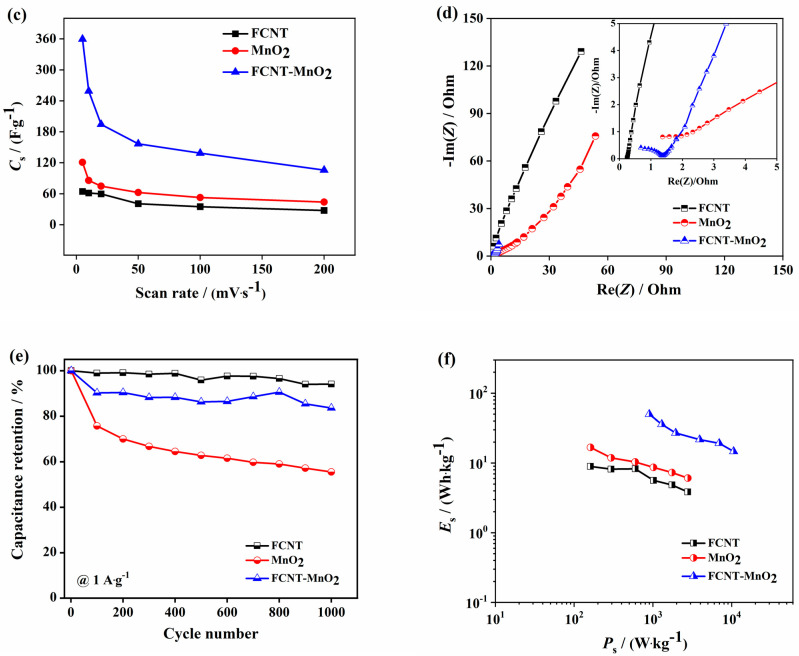
Comparison of electrochemical performance of FCNT, MnO_2_, and FCNT-MnO_2_ in a two-cell configuration with 3 M KOH electrolyte (**a**) cyclic voltammetry at 10 mV·s^−1^, (**b**) galvanostatic charge-discharge curves at 0.5 A·g^−1^, (**c**) specific capacitance from CV, (**d**) EIS-Nyquist plot with inset showing the same in higher frequency region, and (**e**) Cyclic performance up to 1000 cycles from GCD at 1 A·g^−1^, and (**f**) Ragone plot from CV.

The electrochemical analysis was done in a two-electrode system using BioLogic systems. The electrochemical cell was fabricated using a Swagelok cell. Three main techniques were utilized for the electrochemical analysis: cyclic voltammetry (CV), galvanostatic charge discharge (GCD), and potentiometric electrochemical impedance spectroscopy (PEIS or EIS). A comparative electrochemical performance of FCNT, MnO_2_, and FCNT-MnO_2_ is shown in Figure 3. 

In Figure 3a, while FCNT has the least loop area under the curve, FCNT-MnO_2_ has the highest area for the same scan rate. It is also evident that FCNT has EDLC nature with almost rectangular-like structure, while MnO_2_ and FCNT-MnO_2_ have slightly redox-type curves, representing the pseudo-nature of the materials. The redox peaks are seen at 0.35 V, and 0.22 V for MnO_2_, and these are shifted to 0.19 V, and 0.36 V/0.55 V for FCNT-MnO_2_ in the forward and reverse cycles of cyclic voltammetry curve. The presence of peaks indicates the pseudo-nature of the material. The presence of two peaks in the reverse cycle signifies that the ‘Mn’ undergoes multiple valency changes during the electrochemical mechanism [7,10]. This redox mechanism helps in the longer interaction of the electrolyte with the material, thus resulting in high specific capacitance. From the GCD analysis in Figure 3b, the charge-discharge profile appears quite-linear and symmetric with higher charge-discharge time for the hybrid compared to that of the other two owing to its improved charge storage capacity over the individual materials. The plot of specific capacitance vs. scan rate shown in Figure 3c indicates highest values for FCNT-MnO_2_ hybrid among the three and gradually reduces for MnO_2_ and FCNT respectively. The high specific capacitance in hybrid could be due to the fact that MnO_2_ nanoflowers acting as electrolyte reservoir utilizing high effective surface area of the hybrid during the electrochemical mechanism [24]. The specific capacitance for FCNT-MnO_2_ is observed to decrease from 359.54 F g^−1^ at 5 mV s^−1^ to 105.77 F g^−1^ at 200 mV s^−1^ while for MnO_2_ the value is decreased from 120.94 to 43.76 F g^−1^, and in the case of FCNT, it has values of 64.37 and 27.69 F g^−1^ at the aforementioned scan rates, respectively. The EIS analysis in Figure 3d shows the Nyquist plot, in which, the absence of semicircle in the case of FCNT could be due to its non-faradaic reactions and excellent chemical stability of FCNT [12,13]. The nearly straight slope in the low frequency region of Nyquist plot of FCNT-MnO_2_ indicated that the reactions were limited by diffusion resistance (Warburg impedance) rather than charge transfer resistance [24,40]. Also, the straight slope parallel to the imaginary axis indicates perfectly capacitive in nature. The equivalent series resistance (ESR) was found to be 1.37 Ω, and 0.682 Ω for MnO_2_ and FCNT-MnO_2_, respectively. This confirmed that the hybrid material had lesser ESR value than that of MnO_2_ alone proving that the inclusion of carbon nanotube as a conducting path in hybrid nanostructure had an improvement in the overall conductivity. Further, the semicircle developed in the high frequency region of the Nyquist plot (inset of Figure 3d) correspond to the charge transfer resistance (CTR) developed [29]. The value of CTR (calculated using the radius of the semicircle) was found to be 0.82 Ω for FCNT-MnO_2_, however, for MnO_2_ there is no visible semicircle; instead, it shows a flat line nature (inset of Figure 3d) which can be attributed to a large value of ESR existing at higher frequencies. Hence, the addition of FCNT to MnO_2_ had greatly reduced both ESR and CTR of the hybrid nanostructure. The Bode plot obtained from EIS analysis (Supplementary Section—S4.5, S4.6, and S4.7) shows that at the lowest frequency of 0.1 Hz, the negative phase angle was found to be 71.34°, 54.82°, and 66.6° for FCNT, MnO_2_ and FCNT-MnO_2_ respectively. It was observed that FCNT and FCNT-MnO_2_ had phase angles close to capacitor (−90°), and hence FCNT-MnO_2_ can be considered as a good supercapacitor electrode material. It was observed that in the case of MnO_2_, the negative phase angle had drastically increased to 54.82° from 73.97° (Figure S6) indicates that it had very poor conductivity and developed resistance after 1000 cycles. The cyclic performance shown in Figure 3e was evaluated for 1000 cycles for each one. Hybrid FCNT-MnO_2_ showed a higher capacitance retention of 83.59% compared to MnO_2_ with 55.52%. MnO_2_ exhibited poor capacitance retention due to its poor conductivity and development of high charge transfer resistance [41]. The capacitance retention is dependent on the overtime development of charge transfer resistance in the electrode material. Since FCNT is chemically stable compared to MnO_2_ and principally has EDLC nature, it does not develop any significant charge transfer resistance overtime as its electrochemical reactions are non-faradaic. As a result, the capacitance retention is maximum for FCNT whether in case of FCNT-MnO_2_ hybrid, there is evidence of a small charge transfer resistance development due to the presence of MnO_2_ which results in the loss of capacitance retention. The hybrid nanostructured FCNT-MnO_2_ excellent capacitance retention can be attributed to the addition of FCNT to MnO_2_ which improved the stability of MnO_2_ resulting in higher specific capacitance and specific energy. The ‘Ragone plot’ [41], in Figure 3f, shows the high-performance nature of the hybrid material both in terms of specific energy and specific power. It can be seen that FCNT experienced a sudden drop in the specific energy at higher scan rates since FCNT has no greater charge storage capability. The highest value of specific energy was found for FCNT-MnO_2_ which is 49.93 Wh kg^−1^ at 5 mV s^−1^ followed by MnO_2_ and FCNT with 16.79 Wh kg^−1^ and 8.94 Wh kg^−1^ respectively. Thus, EIS and Ragone plot analyses further corroborate the idea of the improved performance of hybrid FCNT-MnO_2_ over its individual identities.

In terms of the electrochemical reaction of FCNT-MnO_2_, mainly the faradaic reaction mechanism happening between MnO_2_ and KOH which can be written as [18,26]:

$${\mathrm{MnO}}_{2}+K^{+}+{\;e}^{-}\;↔\mathrm{MnOOK}$$

**Figure 4:**
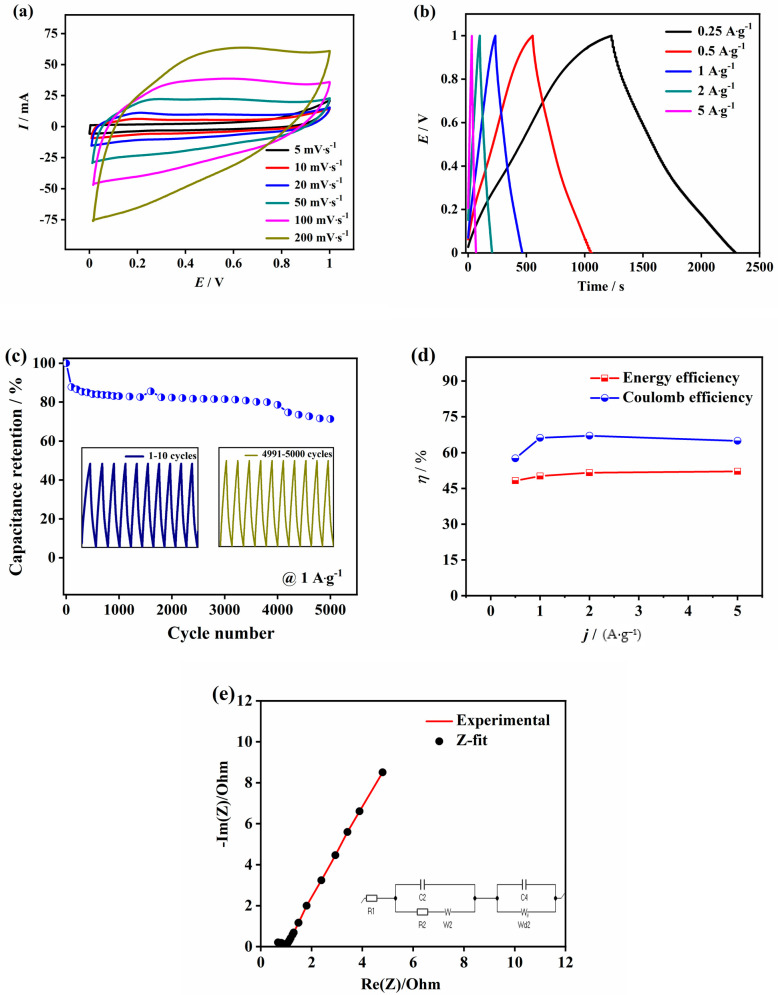
Electrochemical analysis of FCNT-MnO_2_ in a two-cell configuration with 3 M KOH electrolyte (**a**) cyclic voltammetry, (**b**) galvanostatic charge-discharge, (**c**) cyclic performance from GCD, with inset showing first and last 10 cycles of 5000 cycles, (**d**) coulombic efficiency and energy efficiency vs. current density plot, and (**e**) Nyquist plot of experimental data and Z-fit, with its corresponding equivalent circuit.

Figure 4a shows detail CV analysis of FCNT-MnO_2_ at different scan rates from 5 mV s^−1^ to 200 mV s^−1^. The CV analysis shows that as the scan rate increases, the area under the CV curve increases as shown in the Figure 4a. In the hybrid nanostructure, due to the contribution from both faradaic and non-faradaic reactions, the redox peaks were subdued when compared to MnO_2_ alone (Figure S6a). The non-faradaic reactions of FCNT might slightly subdue the pseudo nature of MnO_2_ in the hybrid nanostructure yet the influence of MnO_2_ is reflected in the pseudo-rectangular shape. The GCD analysis at various current densities from 0.25 A g^−1^ to 5 A g^−1^ of FCNT-MnO_2_ is shown in Figure 4b. The charge-discharge profiles appear linear and symmetric, which indicates that the hybrid electrode material is suitable for supercapacitor applications. During the electrochemical mechanism, the MnO_2_ nanoflowers participate in the reaction mechanism and store the electrolyte ions of the electrolyte in between the petals thus enhancing the effective surface area and acting as a reservoir for the electrolyte. The FCNT also stores some of the charge in its hollow tubular structure, thus contributing additional surface area. During electrochemical reactions, the hybrid indicates more charge storage ability without developing much charge transfer resistance as FCNT provide a faster electron transfer path and the charge storage mechanism is mostly done by MnO_2_ nanoflowers through their interaction with electrolyte. A specific capacitance value of 359.53 F g^−1^ was obtained at a scan rate of 5 mV s^−1^ which decreases to 105.77 F g^−1^ at 200 mV s^−1^ with an increasing scan rate as seen earlier. The decrease in the specific capacitance at high scan rates can be attributed to less surface area utilization of the material due to time limited interaction between electrode and electrolyte as the scan rate increases gradually. The equivalent series resistance (ESR) value obtained from the Nyquist plot of EIS analysis (Figure S7a) was 0.678 Ω and 0.682 Ω before and after 1000 cycles, respectively. The semicircle in the Nyquist plot indicates charge transfer resistance (CTR) and its value was found to be 0.685 Ω after 1000 cycles. The phase angle from the Bode plot (Figure S7b) was found to be 59.6° and 66.6° before and after 1000 cycles, respectively. The cyclic performance was evaluated at a current density of 1 A g^−1^ as shown in the Figure 4c and it was found that the cell retained 71.3% of the initial capacitance after 5000 cycles. From the first 10 cycles and the last 10 cycles of total 5000 cycles it is evident that the charge-discharge curves retained the linearity and symmetricity. This observation shows that the hybrid material had retained excellent capacitive features with high energy storage capability even after 5000 cycles. The performance of a material can be understood by evaluating its cyclic efficiency, energy efficiency (η_E_), and coulombic efficiency (η_C_). In case of hybrid supercapacitor due to non-linearity of cell potential, coulombic efficiency (ratio of discharge/charge durations) is overestimated, thus reporting only coulombic efficiency is misleading [56]. Herein, we have reported energy efficiency (the ratio of areas in discharge/charge curves) in parallel with Coulombic efficiency for a more realistic characterization. Finally, the percentage of energy recovered (energy efficiency) was calculated using the following equation

Energy efficiency = (area under the discharging curve/area under the charging curve)%

Figure 4d reports energy efficiency in parallel with coulombic efficiency vs. current density for more practical analysis of FCNT-MnO_2_ hybrid. It is a comparison plot of coulombic efficiency and energy efficiency (percentage of energy recovered) at different current densities. A value of 64.95% and 52.15% of energy efficiency and coulombic efficiency were observed at a current density of 5 A/g for FCNT-MnO_2_ hybrid. Meanwhile, the coulombic and energy efficiency of FCNT and MnO_2_ alone are 65.04%, 62.97%, and 46.84%,48.00% as shown in Figures S5g and S6g, respectively. This indicates a higher value for FCNT-MnO_2_ hybrid compared to MnO_2_ alone and shows the possibility of this hybrid electrode material in practical high current density applications. Figure 4e shows the Nyquist plot after 1000 cycles. It is evident after equivalent circuit fitting using Z-fit software (EC-lab), the following values are noted: electrolyte resistance (R1) = 0.499 Ω, and double layer capacitance (C2) = 37.64 µF, charge transfer resistance (R2) = 0.4051 Ω, pseudo capacitance (C4) = 0.1632 F and the rest of the parameters like Warburg impedance (W–s2) and diffusion resistance (Wd or Rd2) are tabulated along with the results shown in Table 1. 

These results indicate that FCNT-MnO_2_ nanoflower hybrid has improved performance over its individual counterparts. The detailed electrochemical results like specific capacitance, specific energy and specific power for FCNT-MnO_2_ supercapacitor are summarized in Table 2. Additional results are tabulated in the supplementary document (Table S1).

Finally, the hybrid material was tested for its practical application ability with a wider potential window. The electrochemical analysis of FCNT-MnO_2_ was done using organic electrolyte prepared from 1 M TEABF_4_ in acetonitrile. The organic electrolyte provides a wider potential window of 2.7 V suitable for practical applications. A CR2032 coin cell was fabricated inside the glove box under argon inert atmosphere. The results reflected the high energy storage capability of the material as the organic electrolyte provided a wider potential window of 2.7 V compared to that of aqueous electrolyte.

**Figure 5:**
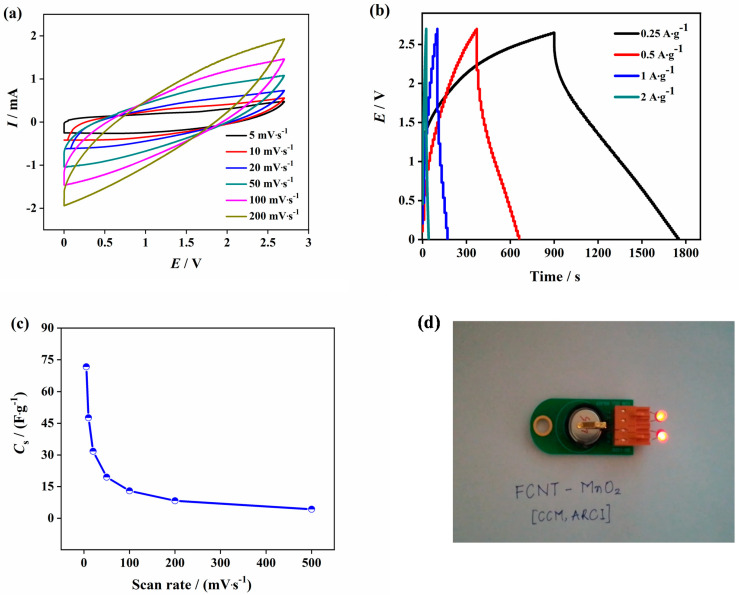
Electrochemical analysis of FCNT-MnO_2_ in the organic electrolyte (1 M TEABF_4_ in Acetonitrile (1:1)) (**a**) CV, (**b**) GCD, (**c**) specific capacitance from CV, and (**d**) LED glowing from FCNT-MnO_2_ cell.

Figure 5a shows the CV analysis with different scan rates. Figure 5b shows the analysis obtained from GCD. The charge-discharge profiles were observed to be slightly asymmetric in nature. This could be attributed to the higher resistance offered form the organic electrolyte and the faradaic reactions lead to non-linear curves at lower current densities. As shown in the Figure 5c, the specific capacitance decreased from 47.62 F g^−1^ at 5 mV s^−1^ to 8.29 F g^−1^ at 200 mV s^−1^. During the electrochemical mechanism, TEABF_4_ in the electrolyte is split into TEA+ and BF_4_- at higher potentials. The positive ions get stored at the negative polarity of the electrode and vice versa. Finally, the cyclic performance of the electrochemical cell with organic electrolyte was tested for 1000 cycles and found that the cell retained above 70% of the initial capacitance after 1000 cycles (Figure S8). One lab-scale prototype CR2032 cell was made using FCNT-MnO_2_ hybrid electrode and organic electrolyte (1 M TEABF_4_ in acetonitrile) as shown in the Figure 5d. It was observed that charging the cell for 2 min resulted in a steady discharge of 4–5 min with almost uniform LED light intensity (visual observation). The detail performance optimization of FCNT-MnO_2_ hybrid electrode and organic electrolyte is going on and will be communicated in future communication. 

## 4. Conclusions

A controlled and repeatable hydrothermal synthesis method was developed to synthesize FCNT-MnO_2_ nanoflower hybrid electrode material for high specific energy supercapacitor application. From the microstructural study, it was confirmed that MnO_2_ nanoflowers are well bonded onto the surface of the functionalized carbon nanotubes, which was essential for reducing the interfacial resistance. This FCNT-MnO_2_ nanoflower hybrid electrode showed a full cell specific capacitance of 359.53 F g^−1^, a specific energy of 49.93 Wh kg^−1^ at 5 mV s^−1^ along with a capacitance retention of 83.59% even after 1000 cycles. This simple, efficient, and economical hydrothermal synthesis technique of the FCNT-MnO_2_ nanoflower hybrid electrode material can be easily scaled up to commercial level for fabricating supercapacitors with high specific energy, specific power, and a long lifecycle. 

## Figures and Tables

**Table 1:** Z-fit parameters of the Nyquist plot of Figure 4e.

Parameter	Value
R1	0.499 Ω
C2	37.64 µF
R2	0.4051
s2	2.182 Ω·s^−0.5^
C4	0.1632 F
Rd2	28.71 Ω
td2	−0.1702 ms

**Table 2:** Electrochemical results of FCNT-MnO_2_ hybrid from cyclic voltammetry.

Cyclic Voltammetry			
Scan Rate (mV s^−1^)	Specific Capacitance (F g^−1^)	Specific Energy (Wh kg^−1^)	Specific Power (W kg^−1^)
5	359.53664	49.93565	898.84161
10	258.95669	35.96621	1294.78343
20	194.3285	26.99007	1943.28503
50	156.63005	21.75417	3915.75133
100	138.65108	19.25709	6932.55382
200	105.77257	14.69063	10,577.25698

## Supplementary Materials

The following are available online at https://www.mdpi.com/2072-666X/12/2/213/s1, Figure S1: FTIR of functionalized carbon nanotubes, Figure S2: XRD and EDS of FCNT and MnO_2_, Figure S3: Morphology of FCNT and MnO_2_, Figure S4: Raman spectrum of FCNT-MnO_2_, Figure S5: Electrochemical analysis of FCNT, Figure S6: Electrochemical analysis of MnO_2_, Figure S7: Electrochemical impedance analysis of FCNT-MnO_2_, Figure S8: Cycling performance of FCNT-MnO_2_ in organic electrolyte, Figure S9: Schematic of two electrode cell configuration, Table S1: Electrochemical results of single electrode of FCNT-MnO_2_ hybrid from galvanostatic charge-discharge. The supplementary document provides detail explanation of FTIR, XRD, EDS, Raman spectroscopy and electrochemical analysis of FCNT and MnO_2_.
