# Supplementary material for: Functionalized Carbon Nanotube and MnO2 Nanoflower Hybrid as an Electrode Material for Supercapacitor Application
Source: Micromachines (Basel). 2021 Feb 20;12(2):213. doi: 10.3390/mi12020213 (PMC7923425; doi:10.3390/mi12020213)
Supplement: Supplementary file 1 [file micromachines-12-00213-s001.pdf]

Article

Functionalized Carbon Nanotube and MnO_2 Nanoflower Hybrid as An Electrode Material for Supercapacitor Application

Sagar Mothkuri, Honey Gupta, Pawan K. Jain, Tata. Narsinga. Rao, G. Padmanabham and Supriya Chakrabarti

S1. FTIR analysis

After the functionalization, the functional groups attached on the surface of nanotubes were identified using FTIR analysis as shown in the Figure S1. The intense peak at 3435.22 cm^{-1} refers to the O-H stretching vibrations of hydroxyl and carboxyl groups. The three other major peaks found in the FTIR spectrum are 1628.61 cm^{-1}, 1380.85 cm^{-1}, and 1101.10 cm^{-1} correspond to C=C, -OH, and C-O functional groups respectively. Other two minor peaks at 2917.57 cm^{-1} and 2845.97 cm^{-1} belong to C-H bonding of carboxyl groups and might be arising from the traces of water in the KBr disk used for the analysis which cannot be fully removed. The peak in the fingerprint region at 604.97 cm^{-1} represents a bending vibration of CH-bond vibrations indicating the surface functionalization by carboxylic group. The overall FTIR analysis indicate that the successful functionalization of carbon nanotubes [1-4].

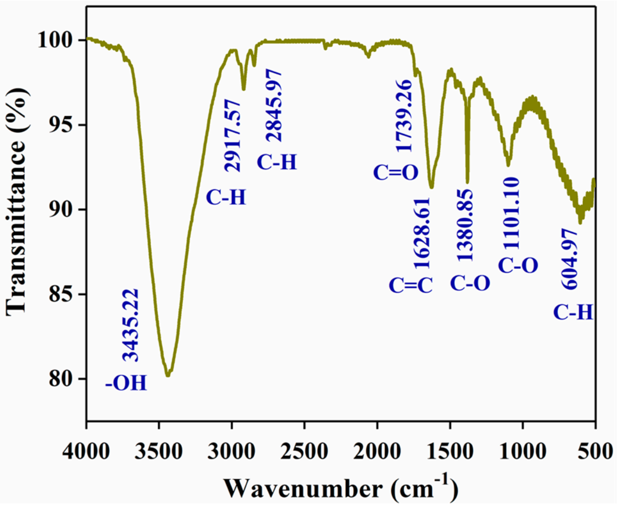

Figure 1. FTIR of functionalized carbon nanotubes.

S2. XRD and EDS analysis

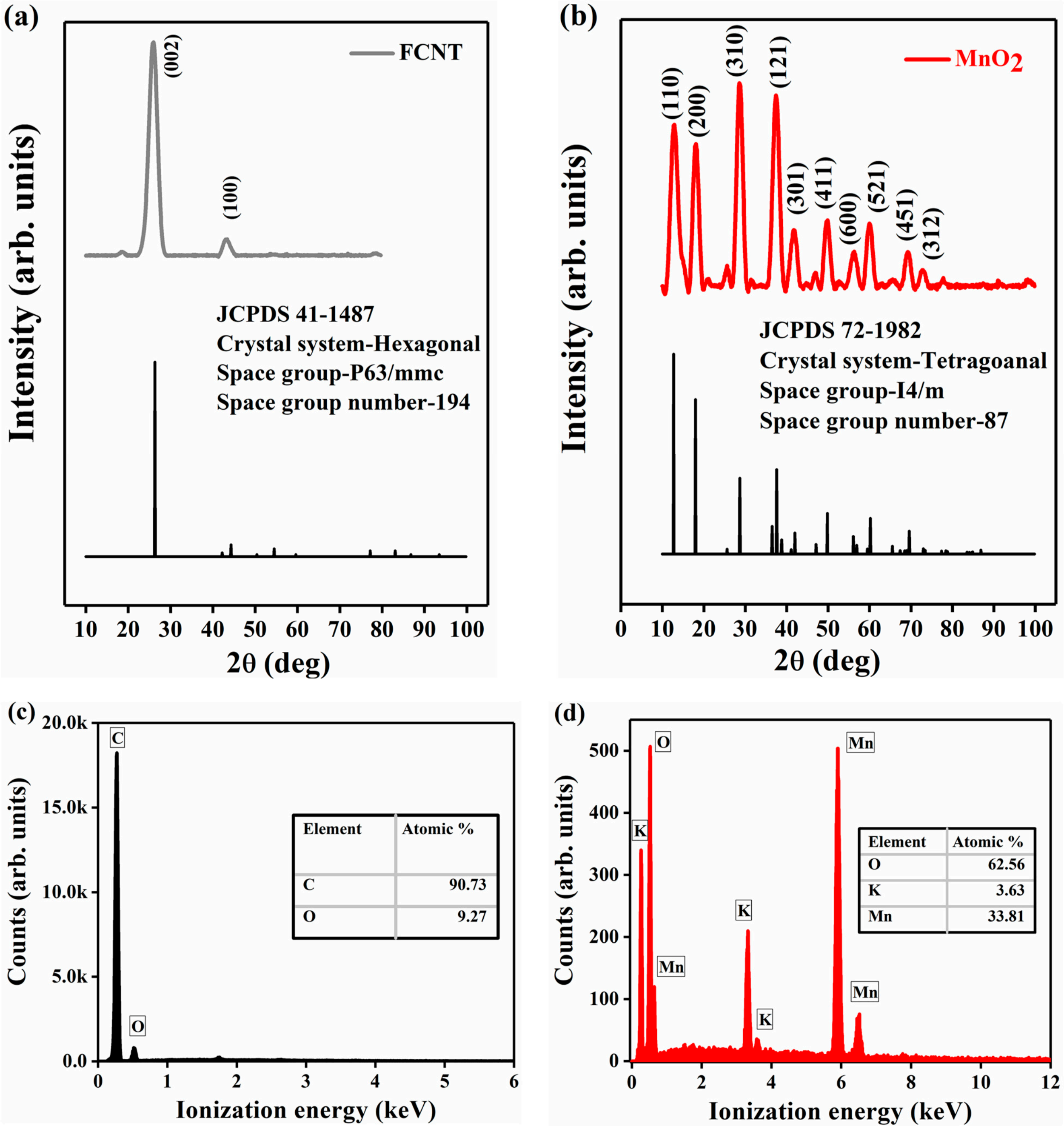

Figure 2. XRD of **(a)** FCNT and **(b)** MnO_2 with their standard JCPDS matching; EDS of **(c)** FCNT and **(d)** MnO_2 with atomic percentages of the constituents.

3. SEM of FCNT and MnO_2

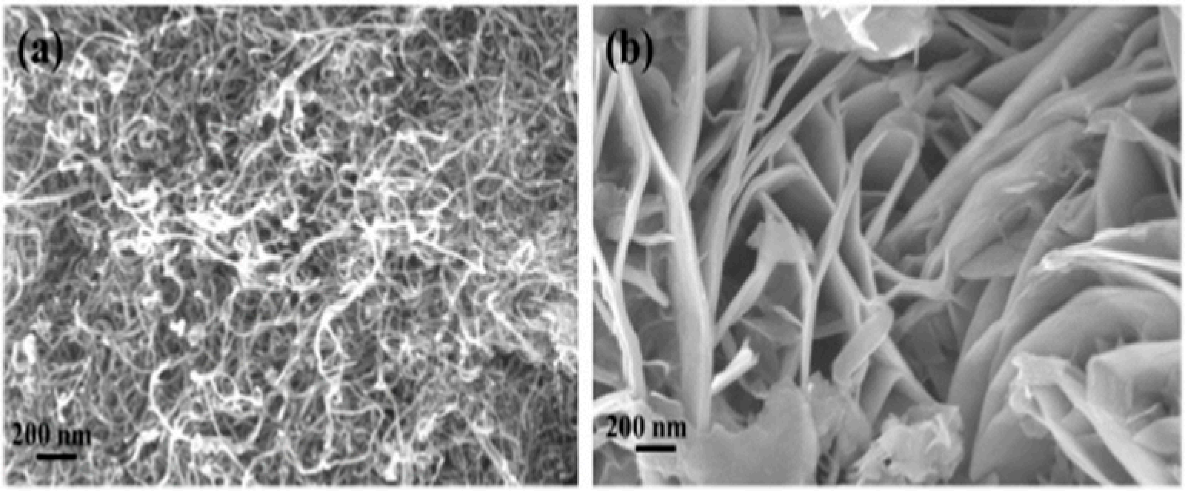

Figure 3. Morphology of **(a)** FCNT and **(b)** MnO_2.

S4. Electrochemical analysis

S4.1. Raman analysis

The Raman spectrum shown in Figure S8 indicates the presence of major bands of CNT and MnO_2. The D-band at 1347.12 cm^{-1} is the most prevalent among all the graphitic compounds, originated from defects and disorder in the carbon structure from the hybridized vibrational modes of the graphene edges. The G-band at ~1588.23 cm^{-1} originates from the stretching of C-C bonds representing sp^2 hybridization and graphitized carbon structure. The 2D-band at 2685.02 cm^{-1} represents the second-order two-phonon process, resulting from the interlayer interactions within the depths of graphene layers of the CNT [9]. The D-band to G-band ratio was found to be 1.56 which represents the defect dominated structure of FCNT. The two major bands at 561.21 cm^{-1} and 637.14 cm^{-1} represent the formation of MnO_2 in which the peak at 637.14 cm^{-1} arises from the symmetric stretching vibrations of the MnO_6 octahedron in the MnO_2 compounds [10]. The band located at 561.21 cm^{-1} can be attributed to the ν_3 (Mn–O) stretching vibration in the basal plane of MnO_6 sheets [11]. This feature is particularly strong in birnessite compounds and related to the high rate of Mn(IV) in the birnessite family. Hence, hybrid FCNT-MnO_2 has the characteristic peaks of both FCNT and MnO_2 with defects dominated FCNT structure.

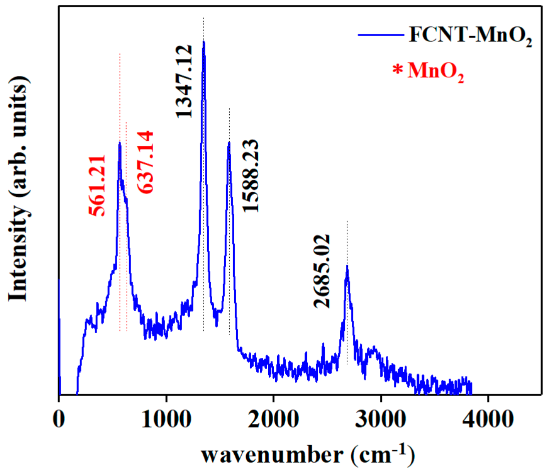

Figure 4. Raman spectrum of FCNT-MnO_2.

S4.2. Cell preparation

The electrochemical cell (full-cell or 2-terminal configuration) was prepared by preparing electrodes at the first stage. The electrode was prepared by taking 8:1:1 weight proportions of active material, carbon black, and binder (PVDF), and thoroughly grinding using a mortar-pestle, with the addition of NMP solvent. The slurry formed was coated on to 35µm thick graphite foil. The electrodes were vacuum dried overnight at a temperature of 90 °C. Graphite foil was chosen as a substrate as it doesn't corrode with KOH electrolyte, whereas metal foils like aluminum (Al) or copper (Cu) or stainless steel (SS) easily get corroded with KOH electrolyte for longer cycle tests. Graphite foil is lightweight, possess good electrical conductivity and less expensive compared to metal foils. The adhesion of the electrode material (FCNT-MNO_2 hybrid) to Al, SS and Cu foils was weak resulted to easy peel off the electrode material from the substrate. However, the adhesion of FCNT-MNO_2 hybrid electrode material to the Graphite foil substrate was strong enough to achieve a stable device structure. Graphite foil has the corrosion resistant property to most of the electrolytes which was another reason for choosing it in our study. The electrodes were weighed before keeping them in a cell for electrochemical analysis. Whatman filter paper wetted with 3M KOH electrolyte was sandwiched between the electrodes prepared and were finally sealed in a commercial Swagelok cell for electrochemical characterization.

S4.3. Analysis parameters

The electrochemical analysis was done using three techniques: cyclic voltammetry (CV), galvanostatic charge discharge (GCD), and potentiostatic electrochemical impedance spectroscopy (PEIS or EIS).

CV is used with input ramp signal of potential 0 to 1 V. The CV curve is plotted between potential (x-axis) and current (y-axis) at different scan rates of 5, 10, 20, 50, 100, and 200 mV s^{-1}. Galvanostatic charge-discharge is used with a static input current. The GCD curve is generated between time (sec) and potential (V) at different current densities of 0.25, 0.5, 1, 2, and 5 A g^{-1}. This technique is also used for the cyclic performance analysis. The third technique used in this analysis is potentiostatic electrochemical impedance analysis (EIS) with an input sine wave of V_{rms} of 5 mV amplitude, applied frequency range between 0.1 Hz to 10 kHz, and with an initial voltage equals to open circuit voltage of the electrochemical cell just before the analysis.

S4.4. Analytical formulae

The specific capacitance (C_s), specific energy (E_s) and specific power (P_s) are calculated both from cyclic voltammetry and galvanostatic charge discharge using the following equations [5-8].

From cyclic voltammetry:

Specific capacitance:

$$C_s = \frac{\int Idv}{V_s \times \Delta V \times m} \quad (1)$$

where C_s is the specific capacitance in F g^{-1}, $\int Idv$ is the integral area under the CV curve in mA V^{-1}, V_s is the scan rate mV s^{-1}, ΔV is the potential window in '*V*', and m is the total mass of active material in the electrochemical cell in 'g'.

Specific energy:

$$E_s = \frac{1}{2} \times C_s \times \Delta V^2 \times \frac{1000}{3600} \quad (2)$$

where C_s is the specific capacitance obtained from Equation 1, and the specific energy, E_s is measured in Wh kg^{-1}.

Specific power:

$$P_s = \frac{1}{2} \times C_s \times \Delta V \times V_s \quad (3)$$

where C_s is the specific capacitance obtained from Equation 1, and the specific power, P_s is measured in W kg^{-1}.

From Galvanostatic charge discharge:

The energy efficiency (η_E) and coulombic efficiency (η_C) are calculated using the following equation (4) and (5) respectively.

$$\eta_E = \frac{\text{Area under discharging curve}}{\text{Area under charging curve}} \times 100\ \% \quad (4)$$

$$\eta_C = \frac{\text{Discharging time}}{\text{Total charge \& discharge time}} \times 100\ \% \quad (5)$$

Specific capacitance:

$$C_s = \frac{I \times \Delta t}{\Delta V \times m} \quad (6)$$

where C_s is the specific capacitance in F g^{-1}, I is the current applied in mA, Δt is the time under discharge curve, ΔV is the potential window in '*Volt*', and m is the total mass of active material in the electrochemical cell in 'gm'.

The specific capacitance of single electrode can be obtained from Eq. (6), as

$$C_s' = 4 \times C_s \quad (7)$$

Specific energy:

$$E_s = \frac{1}{2} \times C_s' \times \Delta V^2 \times \frac{1000}{3600} \quad (8)$$

where C_s' is the specific capacitance obtained from Equation 7, ΔV is the potential window in '*Volt*', and the specific energy, E_s is measured in Wh kg^{-1}.

Specific power:

$$P_s = \frac{E_s}{\Delta t} \quad (9)$$

where E_s is the specific energy obtained from Equation 8, and Δt is the time under discharge curve, and the specific power, P_s is measured in W kg^{-1}.

S4.5. Electrochemical results of FCNT

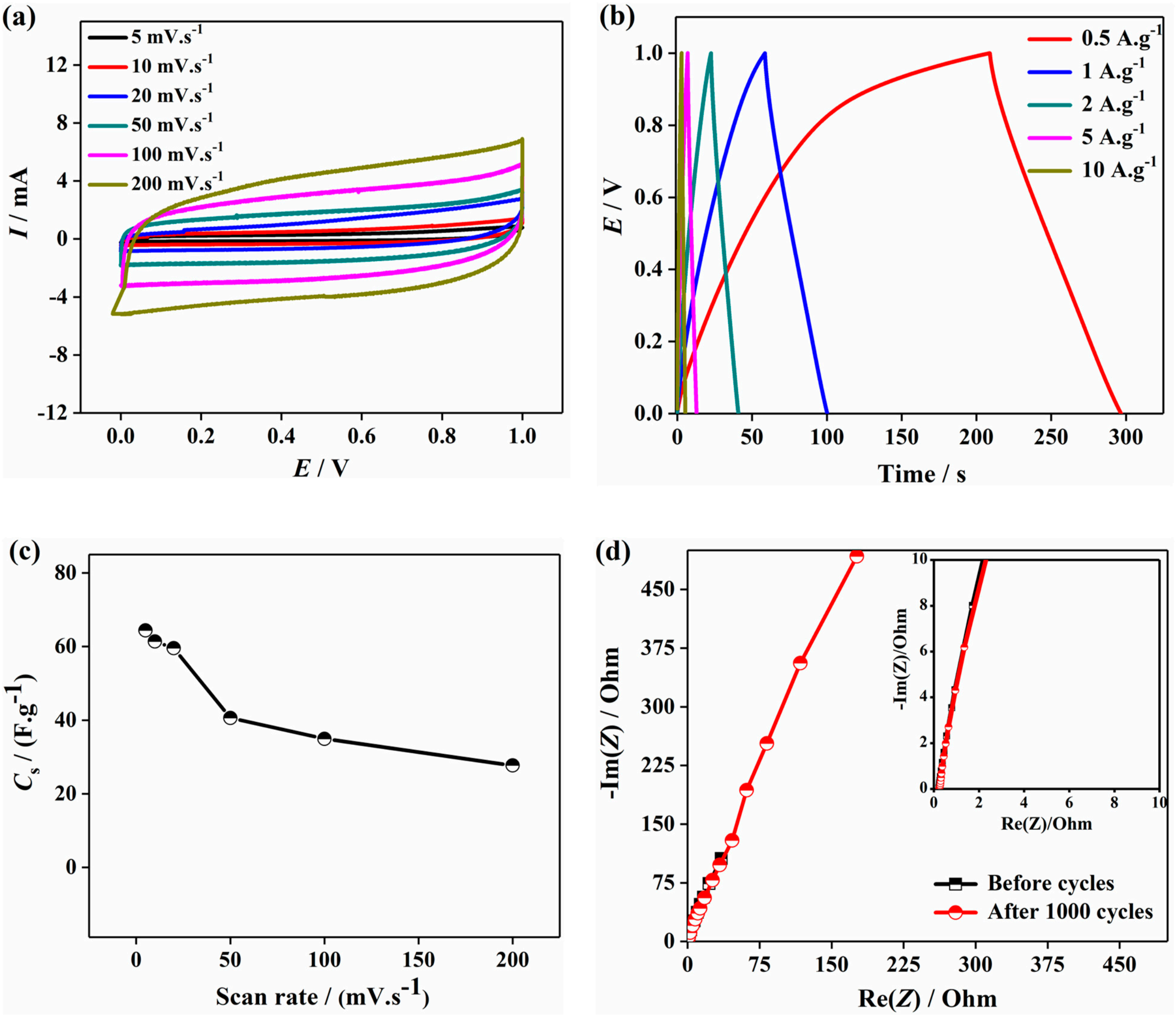

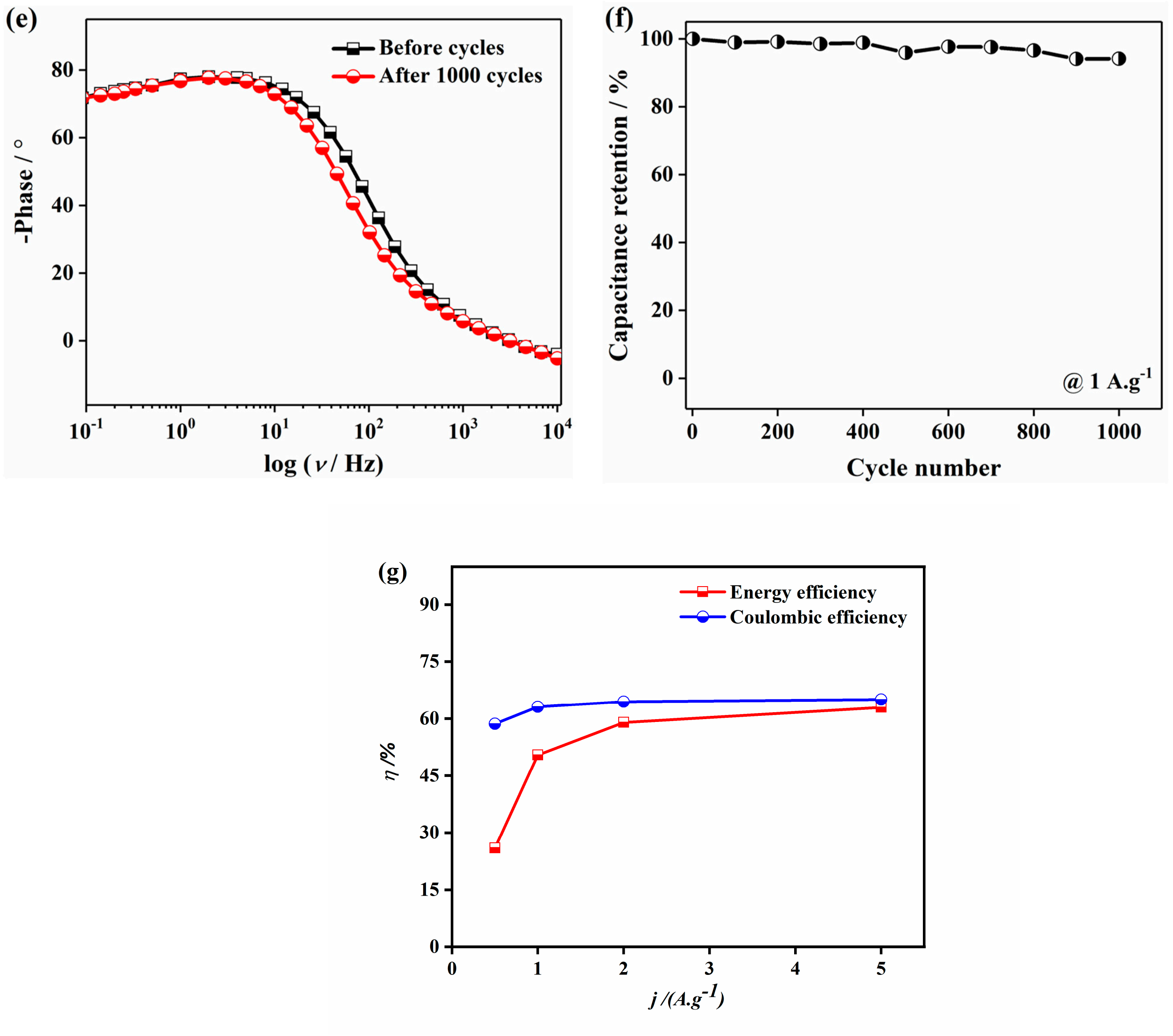

Figure 5. Electrochemical analysis of FCNT **(a)** cyclic voltammetry analysis at different scan rates, **(b)** galvanostatic charge discharge analysis at different current densities, **(c)** plot between specific capacitance and scan rate, **(d)** Nyquist plot (EIS analysis) before and after 1000 cycles with inset showing the plot at higher frequencies, **(e)** Bode plot (EIS analysis) before and after 1000 cycles, **(f)** cycling performance at a current density of 1 A g^{-1} for 1000 cycles, and (g) coulombic efficiency and energy efficiency plot.

S4.6 Electrochemical results of MnO_2

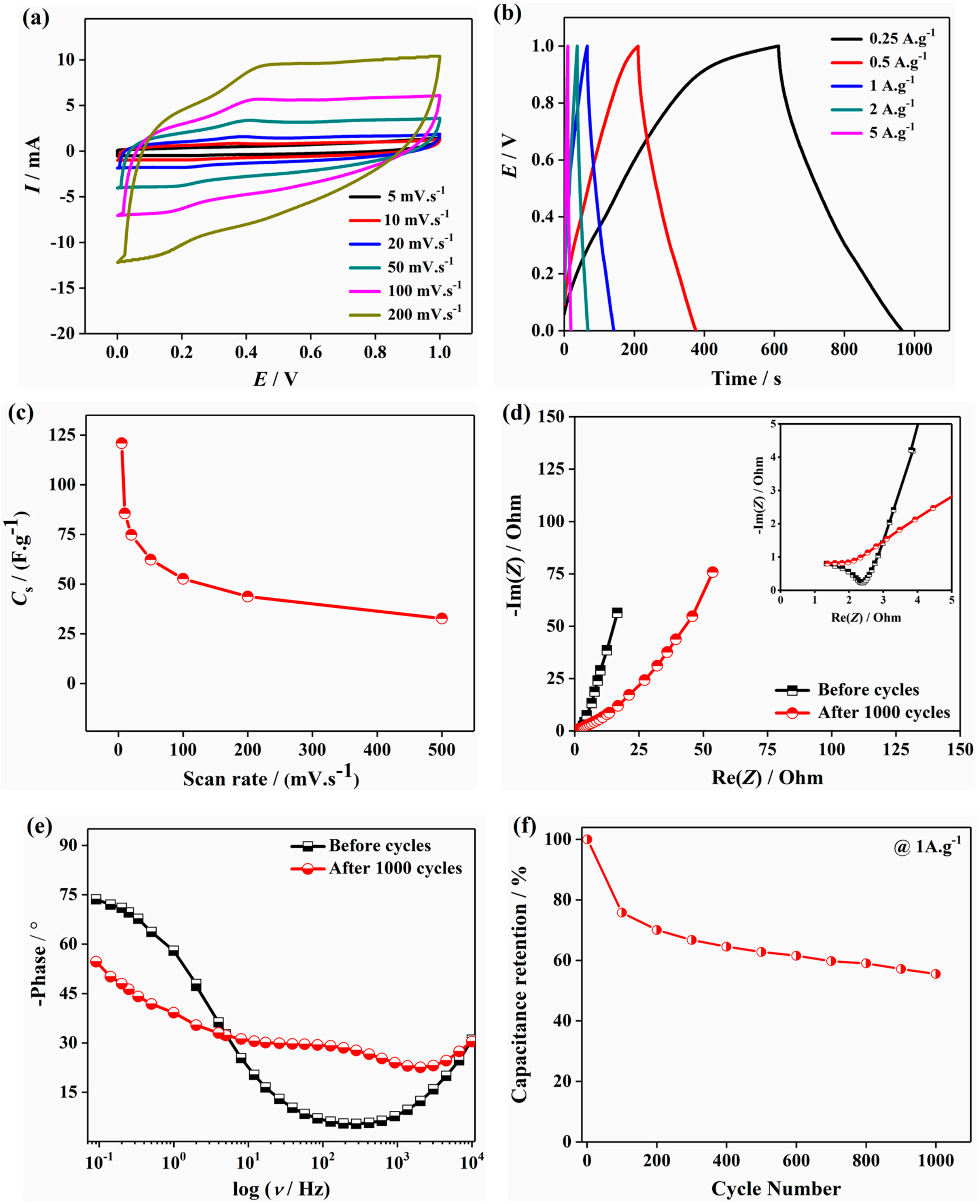

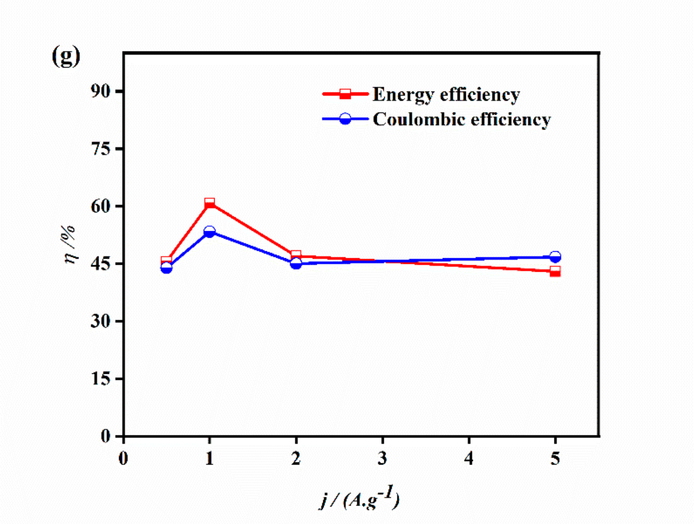

Figure 6. Electrochemical analysis of MnO_2 **(a)** cyclic voltammetry analysis at different scan rates, **(b)** galvanostatic charge discharge analysis at different current densities, **(c)** plot between specific capacitance and scan rate, **(d)** Nyquist plot (EIS analysis) before and after 1000 cycles with inset showing the plot at higher frequencies, **(e)** Bode plot (EIS analysis) before and after 1000 cycles, **(f)** cycling performance at a current density of 1 A g^{-1} for 1000 cycles, and (g) coulombic efficiency and energy efficiency plot.

S4.7. Electrochemical results of FCNT-MnO_2

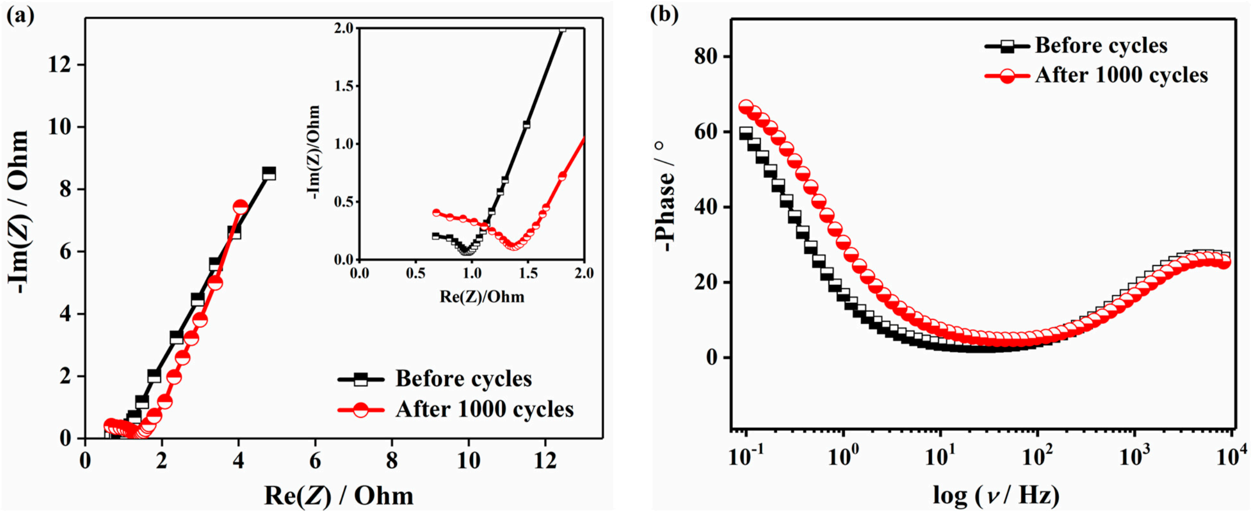

Figure 7. Electrochemical impedance analysis of FCNT-MnO_2 **(a)** Nyquist plot (EIS analysis) before and after 1000 cycles with inset showing the plot at higher frequencies and **(b)** Bode plot before and after 1000 cycles.

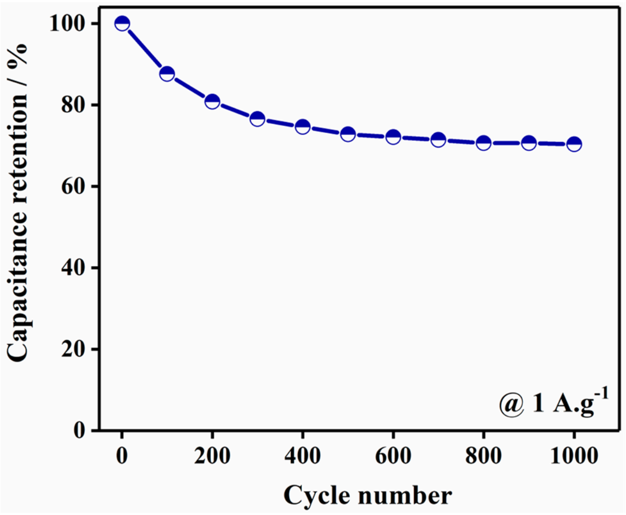

Figure 8. Cycling performance of FCNT-MnO_2 in organic electrolyte (1 M $TEABF_4$ in Acetonitrile (1:1) at a current density of 1 A g^{-1} for 1000 cycles.

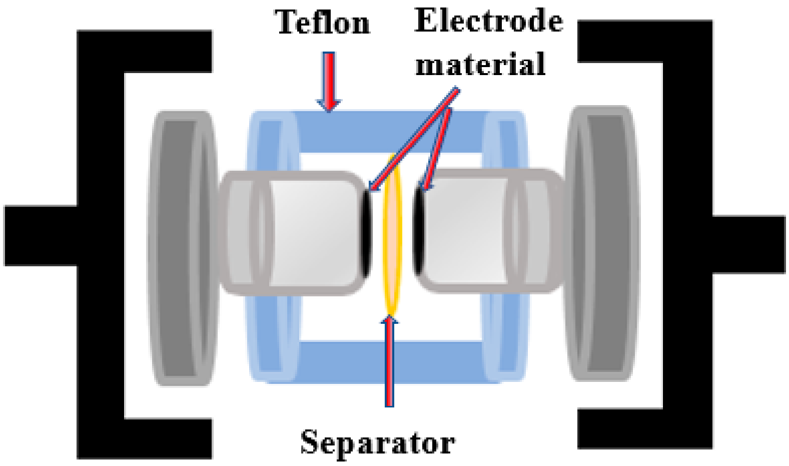

Figure 9. Schematic of two electrode cell configuration.

Table 1. Electrochemical results of single electrode of FCNT-MnO_2 hybrid from galvanostatic charge-discharge. Equations (7)-(9) were used for the calculation of parameters in the current table.

**Galvanostatic charge-discharge**			
**Current density ($A\ g^{-1}$)**	**Specific capacitance ($F\ g^{-1}$)**	**Specific energy ($Wh\ kg^{-1}$)**	Specific power ($W\ kg^{-1}$)
0.25	536.08	74.45556	250
0.5	511.72	71.07222	500
1	466.94	64.85278	1000
2	425.36	59.07778	2000
5	352	48.88889	5000
10	295.4	41.02778	10000

References

1. Balasubramanian K, Burghard M, Chemically Functionalized Carbon Nanotubes, *Small* **2005**, *1*, 180.
2. Furtado CA, Kim UJ, Gutierrez HR, Pan L, Dickey EC, Peter CE, Am J, Debundling and Dissolution of Single-Walled Carbon Nanotubes in Amide Solvents, *Chem. Soc.* **2004**, *126*, 6095.
3. Osorio AG, Silveira ICL, Bueno VL, Bergmann CP, H_2SO_4/HNO_3/HCl—Functionalization and its effect on dispersion of carbon nanotubes in aqueous media, *Applied Surface Science* **2008**, *255*, 2485.
4. Rinzler AG, Liu J, Dai H, Nikolaev P, Huffman CB, Rodriguez MFJ, Boul PJ, Lu AH, Heymann D, Colbert DT, Lee RS, Fischer JE, Rao AM, Eklund PC, Smalley RE, Large-scale purification of single-wall carbon nanotubes: process, product, and characterization, *Appl. Phys. A* **1998**, *67*, 29.
5. Laheaar A, Przygocki P, Abbas Q, Beguin F, Appropriate methods for evaluating the efficiency and capacitive behavior of different types of supercapacitors, *Electrochemistry Communications* **2015**, *60*, 21.
6. Mohan R, Paulose R, Parihar V, Hybrid MnO_2/CNT nanocomposite sheet with enhanced electrochemical performance via surfactant-free wet chemical route, *Ionics* **2017**, *23*, 3245.
7. Li L, Zhong HA, An N, Yu YY, Zhi ML, Hong YW, Facile Synthesis of MnO_2/CNTs Composite for Supercapacitor Electrodes with Long Cycle Stability, *J. Phys. Chem. C* **2014**, *118*, 22865.
8. Sun P, Yi H, Peng T, Jing Y, Wang R, Wang H, Wang X, Ultrathin MnO_2 nanoflakes deposited on carbon nanotube networks for symmetrical supercapacitors with enhanced performance, *Journal of Power Sources* **2017**, *341*, 27.
9. Dresselhaus M.S., Dresselhaus G, Jorio A, Raman Spectroscopy of Carbon Nanotubes in 1997 and 2007, *J. Phys. Chem. C* **2007**, 111, 48, 17887.
10. Tao G, Helmer F, Poul N, A comparison study on Raman scattering properties of α- and β-MnO2, *Analytica Chimica Acta* **2009**, 648, 235.
11. Ogata A, Komaba S, Baddour-Hadjean R, Pereira-Ramos J.-P., Kumagai N, Doping effects on structure and electrode performance of K-birnessite type manganese dioxides for rechargeable lithium battery, *Electrochimica Acta* **2008**, 53, 3084.

© 2021 by the authors. Licensee MDPI, Basel, Switzerland. This article is an open access article distributed under the terms and conditions of the Creative Commons Attribution (CC BY) license (http://creativecommons.org/licenses/by/4.0/).
